# Precision medicine in heritable cancer: when somatic tumour testing and germline mutations meet

**DOI:** 10.1038/npjgenmed.2015.6

**Published:** 2016-01-13

**Authors:** Joanne Ngeow, Charis Eng

**Affiliations:** 1 Cancer Genetics Service, Division of Medical Oncology, National Cancer Centre, Singapore, Singapore; 2 Oncology Academic Clinical Program, Duke-NUS Graduate Medical School, Singapore, Singapore; 3 Genomic Medicine Institute, Cleveland Clinic, Cleveland, OH, USA; 4 Taussig Cancer Institute, Cleveland Clinic, Cleveland, OH, USA; 5 Department of Genetics and Genome Sciences, Case Western Reserve University, Cleveland, OH, USA; 6 Germline High Risk Focus Group, Case Comprehensive Cancer Centre, Case Western Reserve University, Cleveland, OH, USA

## Abstract

Cancer is among the leading causes of death and disfigurement worldwide with an estimated global incidence of 14 million and ~8.2 million cancer-related deaths per annum. An estimated 5–10% of all cancers are hereditary, meaning a single gene mutation contributed to development of the cancer. In other words, inherited cancer has a worldwide incidence of ~1.4 million new cases per annum and a global prevalence of 300 million, and are often poorly recognised. The increase in genetic sequencing capability combined with the decrease in the cost of testing has altered both regulatory policy and clinical oncology practice Well-known examples of clinically important cancer susceptibility syndromes such as those caused by genetic mutations in highly penetrant genes such as *BRCA1/2* hereditary breast-ovarian cancer syndrome genes have provided the framework for the practice of clinical cancer genetics. There is no question that these tests have provided clinical benefit to the patient and her/his family. However, with the expanding role of next generation sequencing in tumour profiling as well as in germline testing, clinicians are now faced with significant new challenges and potentially unexpected opportunities. Issues such as determining how best to deal with gene variants of uncertain clinical significance and the issue of incidental findings of hereditary cancer risk may be encountered during tumour genomic testing will require a concerted effort and dialogue on the part of the broad genomic community.

'Sometimes the first duty of intelligent men is the restatement of the obvious'

- George Orwell, 1938

All cancer is genetic.

From the vantage point of 2015, it is difficult to imagine a time when cancer was not widely accepted as a genetic disease, in the most basic sense of being caused by alterations in the structure and function of genes. And yet, it was not until the latter part of the 20th century that the heritable nature of common cancers started to be widely accepted and actioned.^1,2^

Complex disorders, including breast, ovarian and colorectal cancer, have multifactorial etiologies, including genetic components. Much of what we now know about the signalling pathways involved in these cancers arose from studying Mendelian rules of genetics in which one gene or a few genes results in a hereditary cancer syndrome such as Hereditary Breast and Ovarian Cancer syndrome (HBOC) and Lynch syndrome (LS).

There are over 300 different inherited cancer syndromes. Two well-studied examples include HBOC and LS. Unlike sporadic breast cancer where the average age of onset is 60 years and sporadic ovarian cancer where the mean age at onset is 65, patients with HBOC are characterized by earlier ages of onset of breast and/or ovarian cancer, multifocal and/or bilateral disease, increased risk of male breast cancer as well as a family history of cancers. The majority of HBOC are due to germline mutations in two breast cancer susceptibility genes, *BRCA1* and *BRCA2*. Women within the general population have a 13% lifetime risk of developing breast cancer and a 1% lifetime risk of developing ovarian cancer. For women with *BRCA1/2* mutations, however, the lifetime cancer risk is greater. It is estimated that 47–66% of women with *BRCA1* mutations will develop breast cancer by age 70, while 35–46% of them will develop ovarian cancer by that age. In addition to breast and ovarian cancer, *BRCA2* mutation also confers increased risks of male breast and pancreatic cancers, and melanoma as well. Both *BRCA1/2* mutations associate with prostate cancer risk but without lowering the age-at-risk. Currently, identified germline mutations account for ~5% of colorectal cancer cases. The general population has a 6% lifetime risk of developing colorectal cancer, but for LS patients with mismatch repair gene mutations the risk increases to 80%, and the risk of developing certain other cancers (e.g., endometrial cancer in women) also increases substantially. Individuals with inherited cancer syndromes are also at risk of synchronous and metachronous primary cancers throughout their lives.

Morbidity from these heritable mutations significantly impacts on patients and their families. This is reflected in the US Department of Health and Human Services’ Healthy People 2020 benchmarks. These health care objectives are released every decade using firm evidence-based information regarding cost-effective clinical benefits at the population level. The 2020 objectives included new health promotion areas to concentrate on and for the first time includes genomic medicine in the list of priorities. The genomic objectives of Healthy People 2020 emphasise the importance of obtaining a family and genetic history as a potential and powerful guide for clinical and public health initiatives.

Using nationally representative survey data from over 35,000 women with no personal history of breast or ovarian cancer, only 10% of women who met guideline criteria for hereditary cancer testing (based on family history) reported having discussed the option with a healthcare professional. Further, only 1.41% of these clinically appropriate patients reported having undergone genetic testing.^3^ This was why the first Healthy People 2020 genomic recommendation is that women with a family history of breast or ovarian cancer should receive genetic counselling and set the target of at least a 10% improvement by the end of the decade.^4^ The second genomic recommendation is to increase the number of patients newly diagnosed with colorectal cancer who obtain genetic testing to rule out LS. These genomic recommendations are based on the thought that knowing this information will lead to gene-enabled management and improve the health of involved patients. The genomic agenda of Health People 2020 underscore the importance to identify every single individual who have highly penetrant cancer predisposition syndromes. Both early-detection and risk-reduction strategies save lives for inherited cancer syndromes, in contrast to the situation for the majority of sporadic cancers where such strategies have been woefully unsuccessful. For example, if early-stage colon cancer is detected in LS, total colectomy with ileorectal anastomosis is recommended rather than a segmental/partial colonic resection because of the high risk for metachronous cancers.^5^ In HBOC, women carriers of *BRCA1/2* germline mutations undergo gene-directed high-risk surveillance and are offered prophylactic mastectomy and oophorectomy with the latter resulting in improved overall survival.^6,7^

Because of the LS-related molecular phenotype called mismatch repair deficiency, universal screening for all colorectal and endometrial cancers have proven to be an effective way of addressing Healthy People 2020’s second genomic agenda item.^8,9^ Addressing the first genomic agenda item (heritable breast cancer) is a little harder as there are at least 10 Mendelian high penetrance predisposition genes and not one single clinical or cellular phenotype. This is compounded by having >300 inherited cancer syndromes.^10^ Non-genetics caregivers find it difficult to recognise the clinical ‘red flags’ associated with heritable cancers and hence referrals to clinical cancer genetic services remain haphazard at best.

Every tumour contains inherited (germline) and tumour-specific (somatic) variants. In the last decade, we witnessed dramatic technological improvements in high-throughput sequencing and computational biology, gifting us the ability to amass large amounts of genetic information about both germline and somatic genetic alterations. Our understanding of how these germline and somatic variants contribute towards tumorigenesis have changed the practice of oncology forever. Molecular profiling of tumours allow for targeted therapies which can be safer and more effective than traditional chemotherapies when used in an appropriate molecularly selected patient population. This has been successfully demonstrated for a number of therapeutics targeting the protein products of specific genes that are altered in human cancer, including the use of imatinib in chronic myeloid leukaemias carrying the *BCR-ABL* fusion, erlotinib in EGFR amplified non-small cell lung cancer and vemurafenib in *BRAF*-mutated melanoma. Increasingly, academic and clinical laboratories have moved towards using next generation sequencing panels for such testing on the premise that precision medicine is about matching the right drugs to the right patients. Although this approach is technology agnostic, the tendency to make precision medicine synonymous with genomics is inherently limiting. While we have seen successes, matching therapy to somatic mutation profiles alone is limited by tumour heterogeneity, tumour evolution and our incomplete biological understanding of the relationship between phenotype and cancer genotype.^11–14^

The finding of germline mutations underlying advanced cancers is turning out to be more frequent than had been anticipated.^15–17^ Guidance of how we should deal with incidental findings, which may have clinical significance discovered through such testing, have been addressed by various expert groups such as the American College of Medical Geneticists.^18,19^ While this is not unique to cancer genomics, given the increasing role tumour testing plays in selection of chemotherapy as well as targeted agents, these issues are magnified in contemporary oncology practice.^20^

Although the problem of incidental findings is well-recognised, there are few large systematic studies looking at how best to address this. Catenacci *et al.*,^15^ found that among 110 tumour samples analysed, 21 samples had possible germline mutations in familial cancer genes with only a small subset receiving definitive germline testing. Jones *et al.*^17^ likewise showed in a larger study of 815 tumour–normal paired samples from patients with 15 tumour types. Analyses of matched normal DNA identified germline alterations in cancer-predisposing genes in ~3% of patients with apparently sporadic cancers. In contrast, a tumour-only sequencing approach could not definitively identify germline changes in cancer-predisposing genes and led to additional false-positive findings comprising 31% and 65% of alterations identified in targeted and exome analyses, respectively, including in potentially actionable genes. Collectively, these data suggest that a high fraction of human tumours have alterations that may be clinically actionable and that a small but notable fraction of apparently sporadic cancer patients have pathogenic germline changes in cancer-predisposing genes.

Technically, sequencing and comparison of matched normal DNA to tumour DNA from an affected individual would theoretically allow for accurate identification and subtraction of germline alterations from somatic changes. However, this method is seldom used in cancer diagnostic assays, including next generation sequencing approaches, where only tumour DNA is routinely assessed. In these studies, ~7% (range from 3 to 16%) were believed to carry a germline ‘likely’ pathogenic variant/mutation. While this is to be expected based on past epidemiological studies, or as George Orwell said—obvious. However, it is far less obvious how we should address them in the clinic. Typically, only a small subset of these patients had already been referred to cancer genetics consultation. It is unclear, except for Barton *et al.*^21^ and Funchain *et al*.,^22^ whether the incidental identification of germline mutations led to systematic referral to cancer genetics evaluation. More importantly, perhaps, therefore is the following question: can tumour testing be used to identify clues that could help predict prior probability of finding a germline mutation? Can it be combined with possible corroborative factors such as clinical features, e.g., age of onset, family history or histological features? Funchain *et al*.^22^ identified a mutant allelic fraction threshold of >35% as potentially useful but this will likely be both gene and tumour type specific and may be difficult to generalise.

These studies highlight that the use of tumour-only genomic analyses to infer germline findings is rampant with technical challenges and more importantly significant challenges in correctly identifying patients at risk. As discussed earlier, finding germline mutations for hereditary cancers have clinically actionable implications for both patient and family. Relying on *ad hoc* referral to cancer genetics services has been haphazard with less than perfect ascertainment. The ideal workflow would have all patients receive pre-test genetic counselling prior to surgical resection and tumour testing. This is, however, of limited practical relevance for most centres with limited trained cancer genetics personnel and associated resources.

It is feasible that somatic testing could serve as an additional systematic screen for referral to cancer genetics evaluation and management. We have seen how universal testing for evidence of mismatch repair deficiency in both colorectal and endometrial cancers have allowed clinicians to screen for patients at risk of LS practically and in a manner that is cost-effective.^23–28^ For this to gain traction and to impact on contemporary cancer care, then several things need to occur. First, both the germline and somatic tissue should be analysed. Second, experts who can interpret germline variation need to interpret the germline genomic testing. Germline and somatic variant interpretation utilises different algorithms and differ. Third, genomic tumour boards should include cancer genetic professionals, whether formally trained MD clinical cancer geneticists or cancer genetic counsellors and evolve beyond the present focus on tumour profiling for treatment of metastatic disease. Fourth, interdisciplinary consensus criteria, which comprise demographic, clinicopathologic and/or genomic factors, for referral to cancer genetics evaluation should be standardized for individual tumour types. Finally, cancer genetics clinical services must be adequately resourced to be able to rapidly see such referrals.

As any clinician will affirm, managing a patient with complex medical problems requires multi-disciplinary coordination for best outcomes. Similarly, to tackle the complex genomic enigmas, be it cancer or otherwise, will require the collective expertise of different ‘genomic’ experts (clinicians, scientists and clinician-scientists) looking at the same problem. We hope NPJ-Genomic Medicine will be an unique forum not only for bringing together such discussions but also being the herald of what is to come by challenging her readership to go beyond, if we are to understand the obvious.

'It is a rare mind indeed that can render the hitherto non-existent blindingly obvious.'—Douglas Adams, 1987.
